# Synthetic CRISPR Networks Driven by Transcription Factors via Structure-Switching DNA Translators

**DOI:** 10.1021/jacs.5c06913

**Published:** 2025-06-10

**Authors:** Luca Capelli, Sofia Marzari, Elena Spezzani, Alessandro Bertucci

**Affiliations:** Department of Chemistry, Life Sciences and Environmental Sustainability, 9370 University of Parma, Parco Area Delle Scienze 17/A, Parma 43124, Italy

## Abstract

CRISPR-Cas systems have advanced many domains in life sciences, enabling diverse applications in gene editing, diagnostics, and biosensing. Here, we introduce a platform that leverages transcription factors (TFs) to regulate CRISPR-Cas12a trans-cleavage activity via engineered DNA translators. These dynamic DNA structures respond to TF binding by switching conformations, modulating Cas12a activity. Using TATA-binding protein and Myc-Max as TF models, we optimized DNA translators for precise and tunable control with rapid response kinetics. We demonstrated the platform’s specificity and versatility by integrating TF-induced regulation into synthetic biology networks, including the activation of a fluorogenic RNA aptamer (Mango III) and the creation of an artificial multimolecular communication pathway between Cas12a and Cas13a. This work establishes TFs as effective regulators of CRISPR-Cas systems, enabling novel protein-nucleic acid communication channels, showing potential for novel synthetic biology applications.

## Graphical abstract

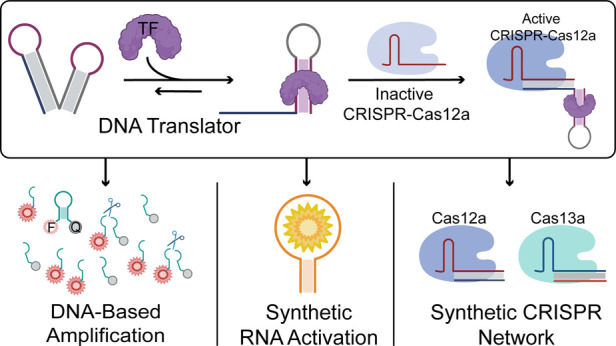

## Introduction

Over the past decade, CRISPR-Cas systems, originally discovered as adaptive immune mechanisms in bacteria and archaea, have enabled transformative applications in gene editing,^1–3^ regulation,^4,5^ imaging^6,7^ and molecular diagnostics.^8–10^ These systems, characterized by RNA-guided target recognition and precise cleavage of nucleic acids, offer unique programmability and flexibility, making them exceptionally powerful molecular tools. The engineering of CRISPR-Cas into a multifunctional platform has made a profound impact on biotechnology, bioengineering, and biomedical research.^11,12^

Among the diverse CRISPR-Cas families, CRISPR-Cas12a has emerged as a particularly versatile tool. Cas12a, a member of the type V CRISPR system, exhibits both target-specific (cis-cleavage) and collateral (trans-cleavage) nuclease activity, enabling precise target recognition and catalytic signal generation.^13–15^ These capabilities make CRISPR-Cas12a a highly efficient tool for gene editing^16^ and biosensing,^17^ and have enabled the development of ultrasensitive analytical assays.^18,19^ One key aspect of advancing CRISPR-Cas systems is achieving precise and programmable control over their enzymatic activities to improve specificity, efficiency, timing, and duration.^20,21^ In Cas12a-based biosensing, various transcriptional and post-transcriptional/translational control techniques have been employed to optimize function,^22,23^ including the engineering of specific formats of crRNA,^24–28^ DNA activators^29–31^ or DNA reporters.^32,33^ In addition, non-nucleic acid inputs, such as light,^34–36^ small molecules,^37–39^ bacteria,^40^ and metal ions,^40,41^ have been explored to regulate Cas12a activity. A promising yet challenging direction involves enabling protein-based regulation of CRISPR-Cas12a, which could significantly broaden its applications across fields beyond traditional nucleic acid targets, opening new avenues in biosensing, diagnostics, and biotechnology. This would enrich biomolecular information processing by creating novel communication channels and artificial signaling pathways. However, since proteins cannot directly activate CRISPR-Cas enzymes, innovative approaches are required to translate protein-derived inputs into nucleic acid signals compatible with CRISPR-Cas machinery. One such strategy leverages the biological activity of target proteins, such as their enzymatic activity or binding affinity for specific ligands, to generate inputs for activating or regulating the Cas12a effector. For example, Cas12a-based detection has been applied for monitoring the enzymatic activity of nuclease, kinase, telomerase or glycosylase.^42–48^ Proteases have also been utilized to cleave peptides that block either the DNA activator or Cas12a itself,^49–52^ thereby controlling the activation of the system. Recently, our group developed a Cas12a-based assay for the MMP2 metalloproteinase, using a peptide-PNA translator activated by MMP2’s enzymatic activity.^52^ Proximity-based mechanisms have further been explored to regulate Cas12a activation, including using antibodies as target elements.^53–55^ The use of DNA-binding proteins like transcription factors (TFs) for Cas12a activation remains relatively unexplored, with only few studies to date employing TFs—involving the p50 subunit of nuclear factor kappa-B (NF-kB)—and allosteric TFs.^56–58^ These prior examples predominantly center on sensor applications of CRISPR-Cas12a, highlighting significant opportunities to expand and enhance the use of this technology.

In this study, we focus on TFs as protein-based regulators of CRISPR-Cas12a nuclease activity. TFs are DNA-binding proteins that play a vital role in biological processes by regulating gene expression through recognizing and binding to specific dsDNA sequences, called consensus sequences.^59^ Previous work has utilized TFs in DNA nanotechnologies, where their natural DNA-binding activity triggers conformational changes in dynamic DNA structures, enabling applications in sensing, imaging, and DNA-based computation.^60–63^ Building on this, we hypothesized that TFs could be harnessed to regulate Cas12a activity by designing structure-switching DNA translators capable of converting TF binding into programmable DNA-based inputs recognized by Cas12a. This approach would create an innovative artificial communication system between CRISPR-Cas12a and transcription factors, expanding the versatility of CRISPR-based tools. Here, we present a library of engineered DNA translators that regulate CRISPR-Cas12a activity in response to different transcription factors. We demonstrate that it is possible to tune the system by rationally designing DNA translators to optimize signal-to-noise ratios and achieve TF concentration-dependent control of Cas12a activity. Furthermore, we show that TF-mediate regulation of Cas12a can be integrated into synthetic biology networks, modulating downstream activation of a functional RNA aptamer and regulating a second, non-naturally related molecular complex such as CRISPR-Cas13 (Figure 1).

**Figure 1:**
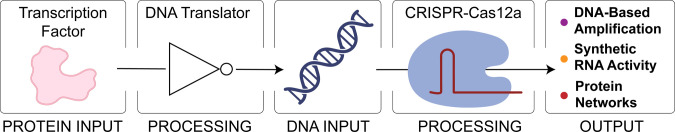
Schematic representation of transcription factor-driven regulation of CRISPR-Cas12a activity, mediated by an engineered DNA translator, enabling execution of different biomolecular operations.

## Results and Discussion

### Rational Design of Different DNA Translators for TBP

The DNA translators reported in this work are sequences that can reversibly switch between two structural conformations in equilibrium, with this balance shifting in response to interactions with specific target elements, such as ligand binding. We designed and engineered TF-responsive DNA translators that incorporate both a consensus sequence for a specific TF and a DNA activator sequence for Cas12a. These DNA translators can adopt two distinct conformations: an OFF state (nonbinding) and an ON state (binding-competent), which exist in dynamic equilibrium. To start, we designed DNA translators responsive to TATA-binding protein (TBP), a ubiquitous transcription factor.^64^ In the thermodynamically more-favorable OFF state, the ssDNA forms two stem-loop structures, sequestering the TBP consensus sequence (purple regions, Figure 2a) and the Cas12a DNA activator (blue region, Figure 2a) within the loops and one of the two stems, respectively, making them inaccessible for recognition. In contrast, the less-favorable ON state presents a single stem-loop structure, positioning the TF consensus sequence within the stem and the DNA activator at the structure’s tail. Upon TF binding to the double-stranded DNA stem containing its consensus sequence, the equilibrium is expected to shift toward the ON state through a population-shift mechanism.^60,65^ The thermodynamic switching constant (*K*_s_) of the DNA switch governs this TF-induced conformational transition. In the ON state, the DNA activator is free to interact with Cas12a, thereby initiating its trans-cleavage activity on FRET-labeled DNA hairpins and generating an amplified fluorescence signal (Figure 2a). The length of the loops, as well as the length of the stems, the relative GC/AT content and the relative position and distribution of the nucleotides in each structure were the key parameters in the design of the translators (see sequences in the Supporting Information). By varying these parameters, we could control the K_s_ of the DNA translator determining the TBP-induced conformational transition and then its interaction with Cas12a. To optimize the regulation of Cas12a activation, we tested several designs of TBP-responsive translators (TBP-Translators) with varying predicted switching equilibrium constants (K_s_), from 0.23 to 0.005. First, we focused on a “Right Tail Design” (RTD), engineering five different TBP-Translators with distinct K_s_, where the DNA activator sequence for Cas12a was sequestered in the right stem of the OFF-state conformation (Figure 2b). Each TBP-Translator was tested both in the presence and absence of the TBP input.

**Figure 2:**
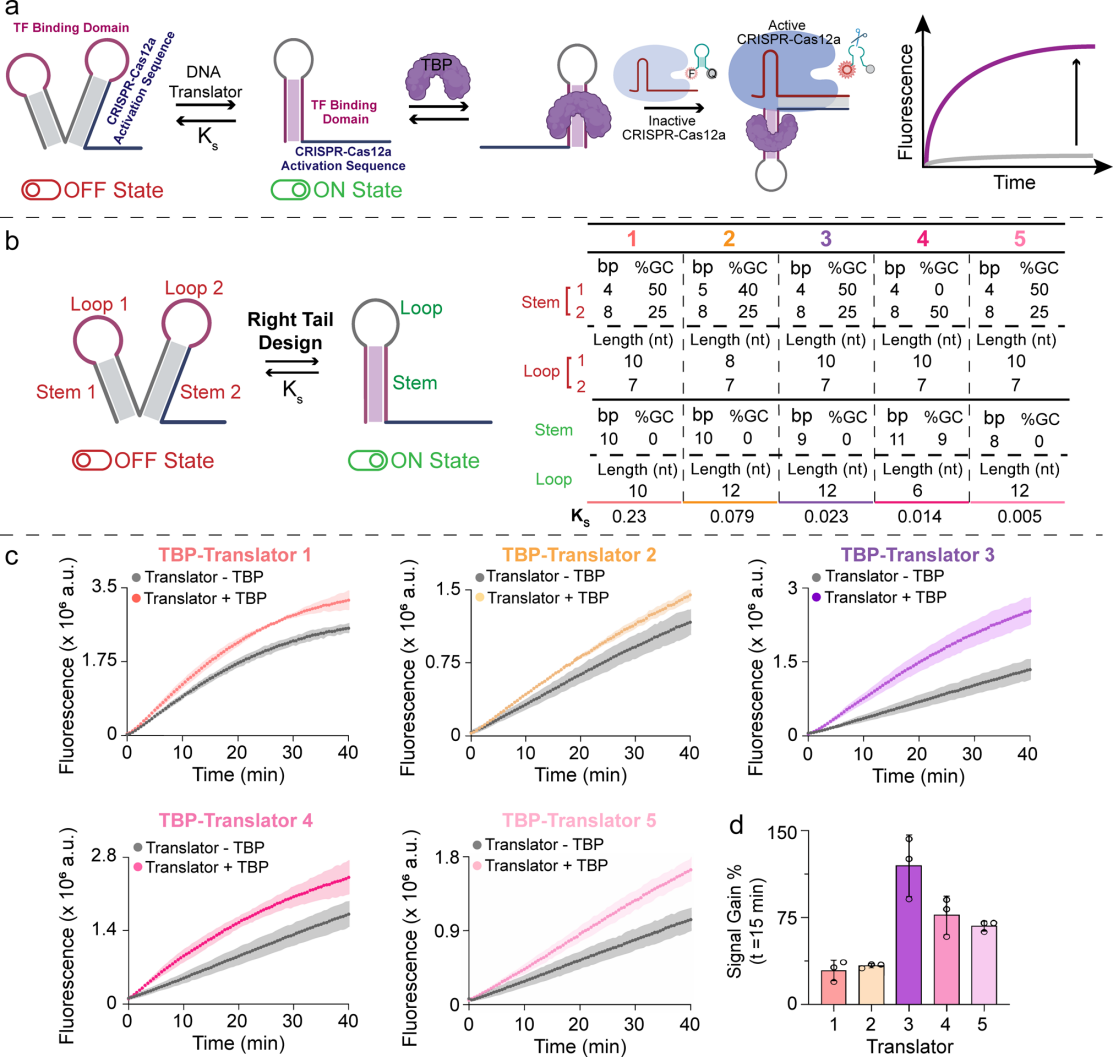
(a) Schematic illustration of the proposed TBP-driven regulation of Cas12a activity mediated by a “right tail” TBP-Translator. (b) Sequence and structural parameters of the stem and loop domains of the five TBP-Translators of the Right Tail Design (bp = base pairs, nt = nucleotides). (c) Fluorescence kinetic profiles of CRISPR-Cas12a trans-cleavage activity triggered by the right tail TBP-Translators in the presence (Translator + TBP) or absence (Translator – TBP) of TBP (20 nm). (d) Signal gain % obtained for each TBP-Translator after 15 min from the start of Cas12a trans-cleavage activity, calculated with the following formula: signal gain % = (Fluorescence Signal – Background)/Background × 100, where the Background is the signal observed when conducting the assay in the absence of TBP (*n* = 3, mean ± SD).

The results, shown in Figure 2c, highlight signal variations for each different K_s_ of the translators. Each translator exhibits differences not only in K_s_ values but also in sequence-specific structural features that influence its interaction with the crRNA–Cas12a complex and the subsequent enzymatic activation. To quantify Cas12a activation, we calculated the signal gain percentage (SG %), defined as the difference between the specific signal recorded in the presence of TBP and the nonspecific background signal (Figure 2d). Among the tested RTD designs, TBP-Translator 3, with a K_s_ of 0.023, exhibited the highest SG % of 120% within 15 min. To confirm this result, we also calculated the ratio between the slopes of the kinetic profiles at the early stage of Cas12a activation in the presence and absence of TBP (Figure S1a). Harnessing the versatility of the translator model, we set out to explore a second design, a “Left Tail Design” (LTD). In this design, the DNA activator sequence for Cas12a was sequestered in the left stem of the OFF-state conformation (Figure 3a,b). We followed a similar approach and engineered five different TBP-Translators with varying K_s_. Through a rational design of the different structures, we could finely regulate these K_s_ values ranging from 0.21 to 0.003 (Figure 3b). The results of the Cas12a-based fluorescence signals in response to the presence of TBP are shown in Figure 3c for each of the five LTD translators. The highest SG % value of 124% was observed for TBP-Translator III, which has a K_s_ of 0.024 (Figure 3d and Figure S1b). We note that the presence of background signal from Cas12a activity in all the tested designs suggests that partial activation of Cas12a is unavoidable under the current molecular design, involving the intrinsic switching equilibrium of the translators and sequence-specific interactions with the crRNA–Cas12a complex.

**Figure 3:**
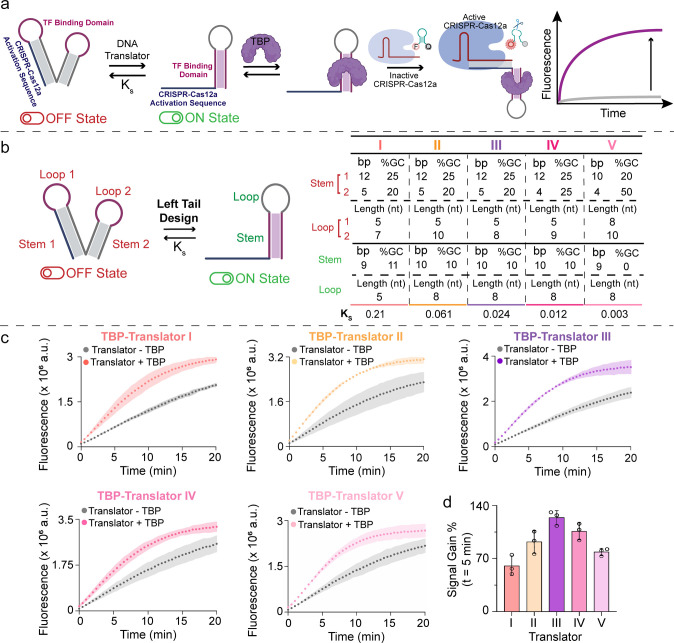
(a) Schematic illustration of the proposed TBP-driven regulation of Cas12a activity mediated by a “left tail” TBP-Translator. (b) Sequence and structural parameters of the stem and loop domains of the five TBP-Translators of left tail design (bp = base pairs, nt = nucleotides). (c) Fluorescence kinetic profiles of CRISPR-Cas12a trans-cleavage activity triggered by the left tail TBP-Translators in the presence (Translator + TBP) or absence (Translator – TBP) of TBP (20 nm). (d) Signal gain % obtained for each TBP-Translator after 5 min from the start of Cas12a trans-cleavage activity (*n* = 3, mean ± SD).

The above results indicate that both translator families exhibit similar behavior: Translator 3 (RTD) and Translator III (LTD), which have nearly identical K_s_ values, led to obtaining the higher SG % responses around 120%. However, a primary difference lies in their kinetic performance. For RTD, the maximum SG % was achieved in 15 min, whereas LTD was faster, reaching its maximum SG % in just 5 min (Figure S2). One factor that may contribute to the observed differences between RTD and LTD is the short unhybridized DNA sequence present at the 3′ end of RTD. This region is partially complementary to the crRNA, potentially facilitating a more efficient transition from the OFF to the ON state in RTD. This could explain the higher signal leakage observed during the early stages of Cas12a activation for RTD compared to LTD. Steric constraints may also play an important role. A previous study^61^ showed that, in similar systems involving transcription factor-controlled DNA strand displacement reactions, the transcription factor remained bound to the DNA translator. This suggests that the full TF–Translator complex might interact with the crRNA–Cas12a complex in this case as well, and that such interactions could vary depending on the structural configuration of RTD versus LTD. Based on its advantageous kinetic performance, which can be highly relevant for TF biosensing applications, we selected TBP-Translator III for a further characterization of the system. By varying the concentration of TBP within a 0.02–100 nM range, we demonstrated dynamic modulation of Cas12a activity in response to the protein concentration (Figure 4a). The calculated dynamic range (defined here as the TF concentration range in which we obtain signals between 10% and 90% of the maximum signal)^66,67^ is between 2 and 40 nM. Next, we performed specificity and inhibition assays. In the specificity assay, we aimed to confirm the specific recognition of TBP by TBP-Translator III, investigating potential cross-reactivity. Using transcription factors such as Myc-Max, EGR1, and EGR4—each recognizing consensus sequences distinct from that of TBP—we observed signals significantly lower than that of TBP (Figures 4b and S3), with only Myc-Max inducing some cross-reactivity signal, approximately one-third of the SG % obtained for TBP. To validate the mechanism for TBP-induced Cas12a activation, we conducted a competitive inhibition experiment. In this experiment, TBP was preincubated with an excess of a stable DNA hairpin (TBP inhibitor, Figure 4b), prior to exposure to the TBP-Translator III. This procedure completely suppressed Cas12a trans-cleavage activity (Figures 4b and S4), providing evidence for the necessity of TBP DNA-binding to induce the activation mechanism.

**Figure 4:**
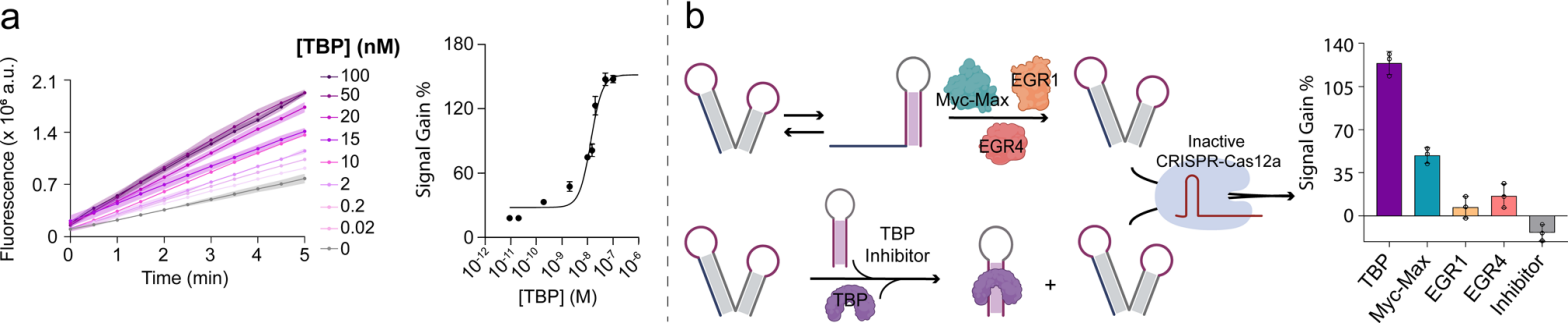
(a) Regulation of Cas12a trans-cleavage activity mediated by TBP-Translator III in the presence of different concentrations of TBP. (Left) Fluorescence kinetic profiles (*n* = 3, mean + SD); (right) binding curve obtained for TBP concentrations varying from 0.02 to 100 nM (*n* = 3, mean + SD). (b) Signal gain % values for specificity tests using TBP-Translator III with nonspecific proteins (Myc-Max, EGR 1 and EGR 4), and inhibition test in the presence of a saturating concentration of TBP inhibitor (*n* = 3, mean + SD).

### Rational Design of a DNA Translator for Myc-Max

Expanding on the LTD of the TBP-Translator, we then designed a new DNA translator for a different transcription factor to demonstrate the generality of our approach. We selected Myc-Max, an additional transcription factor that is clinically relevant in oncology.^68^ Following the results obtained with the TBP-Translators, we applied analogous rational design principles to optimize the stem-loop composition and length for the Myc-Max translator. Our goal was to achieve a switching constant K_s_ comparable to that of the TBP-Translator III (K*s* = 0.023, Figure 5a). A fluorescence kinetic experiment was conducted to assess the activation of Cas12a trans-cleavage activity mediated by Myc-Max binding to the translator (Figure 5b). The highest SG % of 100% was achieved within just 5 min. Similarly to the TBP system, we varied the Myc-Max concentration over a range of 2–200 nM, demonstrating dynamic control over Cas12a activation (Figure 5c) with a dynamic range between 10 and 90 nM. To test cross-reactivity, we incubated the Myc-Max-Translator with TBP, EGR1, and EGR4 and recorded fluorescence kinetic profiles (Figure S5). The results showed negligible cross-reactivity (Figure 5d). Furthermore, preincubating Myc-Max with an excess of a DNA hairpin (Myc-Max inhibitor, Figure 5d) containing the Myc-Max consensus sequence effectively inhibited Cas12a activation induced by Myc-Max (Figure 5d and S6).

**Figure 5:**
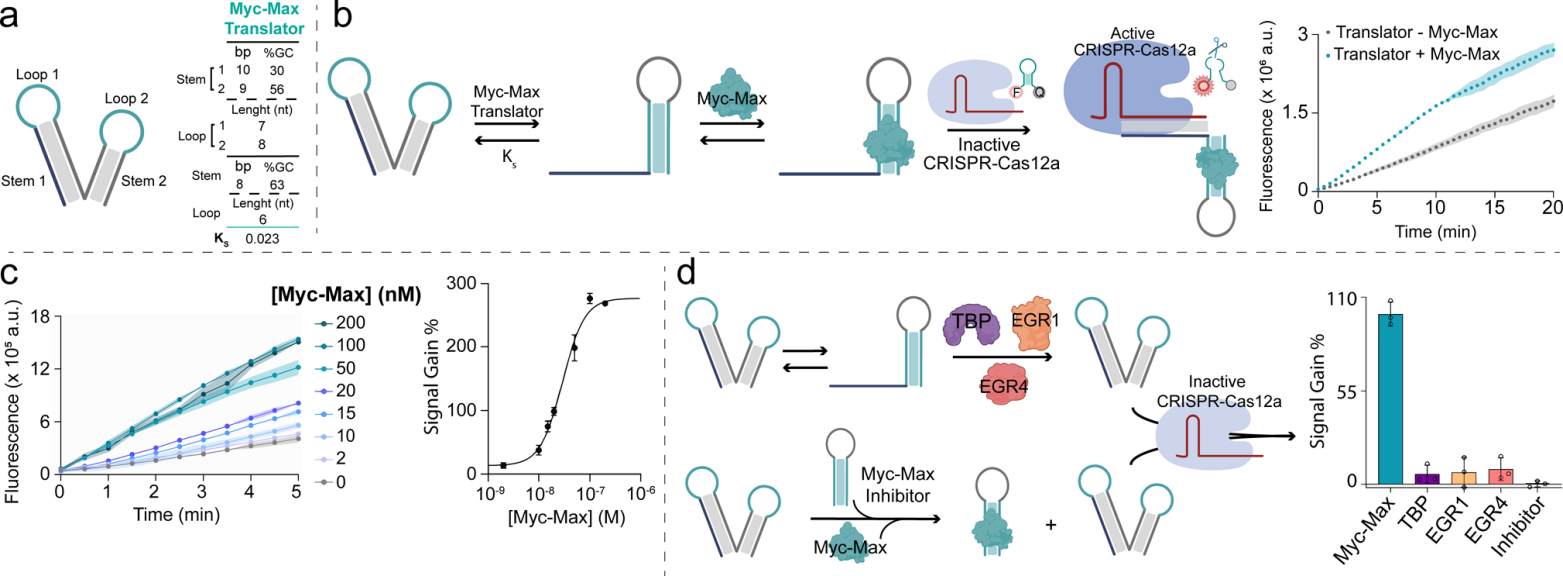
(a) Sequence and structural parameters of the stem and loop domains of the Myc-Max-Translator (bp = base pairs, nt = nucleotides). (b) Schematic illustration of the proposed Myc-Max-driven regulation of Cas12a activity mediated by a “left tail” Myc-Max-Translator, and its fluorescence kinetic profile. c) Regulation of Cas12a trans-cleavage activity mediated by Myc-Max -Translator in the presence of different concentrations of Myc-Max. (Left) Fluorescence kinetic profiles (*n* = 3, mean + SD); (right) binding curve obtained for Myc-Max concentrations varying from 2 nM to 200 nM (*n* = 3, mean + SD). (d) Signal gain % values for specificity tests using Myc-Max -Translator III with nonspecific proteins (TBP, EGR 1 and EGR 4), and inhibition test in the presence of a saturating concentration of Myc-Max inhibitor (*n* = 3, mean + SD).

### Activation of RNA Mango via TF-Driven Cas12a Activity

Having successfully achieved precise control over the TF-driven regulation of Cas12a trans-cleavage activity, we set out to explore integrating this mechanism into more complex biomolecular networks. This capability would further demonstrate the platform’s potential to support sophisticated DNA-based and CRISPR-Cas-based computing. To start, we focused on the controlled activation of a functional RNA structure (Figure 6a). Aptamers are oligomers that exhibit specific binding activity toward target molecules.^69^ While numerous studies have demonstrated the use of aptamers to regulate Cas12a activation or employed Cas12a for aptamer degradation and small molecule release,^70–72^ controlling the release and activation of an aptamer by using Cas12a remains unexplored. Developing such a strategy would significantly enhance the synthetic biology toolbox. Mango III, a synthetic fluorogenic RNA aptamer, was selected for this- purpose due to its ability to emit a strong fluorescence signal upon binding to the thiazole orange (TO-1) dye.^73,74^ By employing a blocking DNA strand that partially hybridizes to the TO-1 recognition sequence of the aptamer, we were able to prevent the RNA aptamer from folding into its optically active conformation (Figure S7). When the blocking strand hybridizes to Mango III, it creates a DNA bulge that can be cleaved by active Cas12a. This cleavage event produces two shorter DNA fragments, which are unable to maintain Mango III in its blocked state, allowing it to fold into the correct conformation and restore fluorescence upon TO-1 binding (Figure 6b). To control this system, we utilized TBP-Translator III or Myc-Max-Translator. In both cases, we varied the concentration of the TF within a range of 0 to 20 nM. As shown in Figures 6c-6d, the TF-modulated trans-cleavage activity of Cas12a enables to finely regulate the activation of Mango III. This result confirms the successful establishment of an artificial communication pathway involving a transcription factor, a CRISPR-Cas12a complex, and a synthetic RNA. These protein–nucleic acid circuits hold potential for applications in bioregulation, cancer therapy, the control of biochemical processes, and beyond.^75–78^

**Figure 6:**
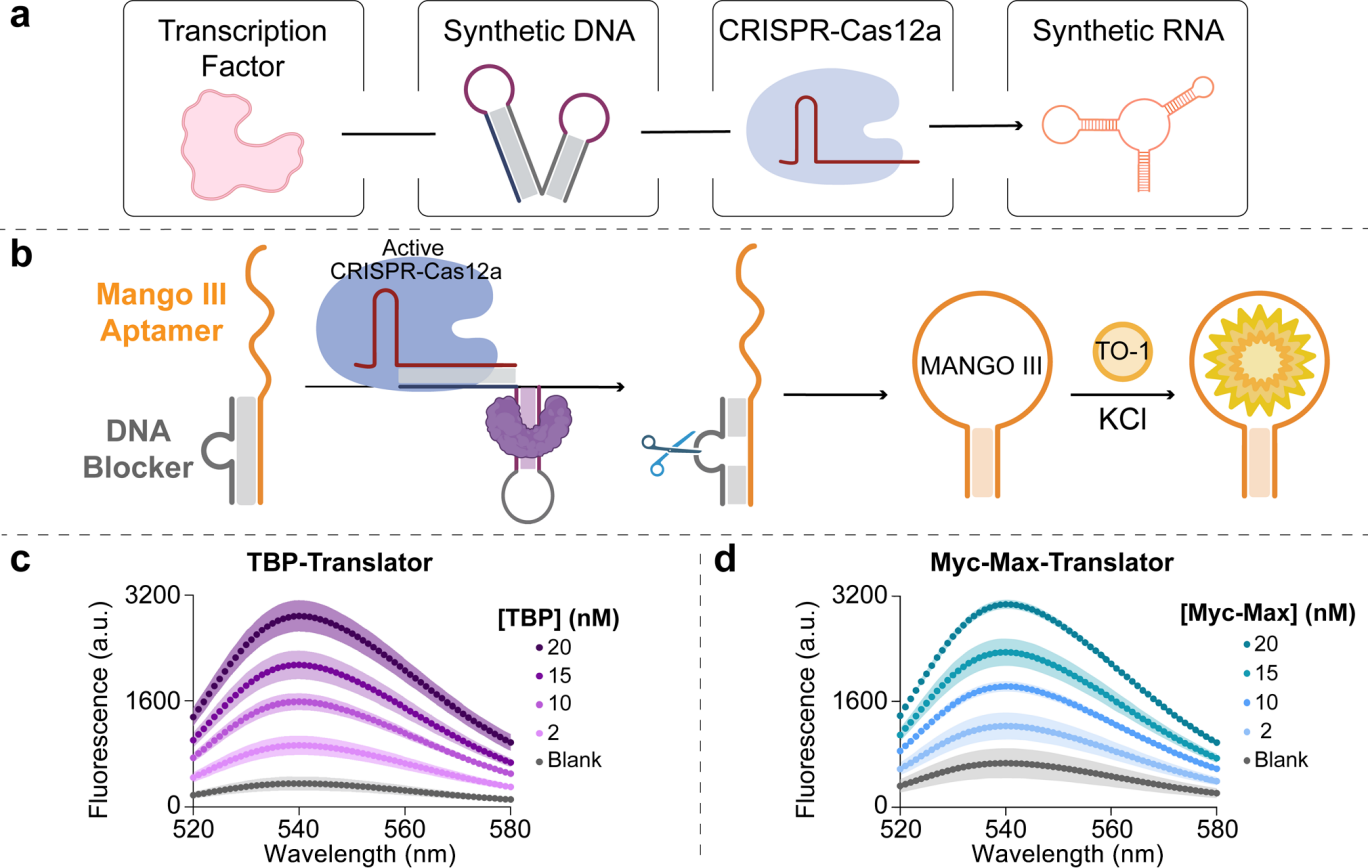
(a) Schematic representation of the molecular network modulated by the interaction between a transcription factor and its DNA translator to regulate CRISPR-Cas12a activity for the subsequent activation of a synthetic RNA aptamer. (b) Schematic representation of Mango III aptamer activation controlled by TF-induced CRISPR-Cas12a trans-cleavage activity. (c) Regulation of Mango III aptamer fluorescence mediated by TBP-Translator III in the presence of different concentrations (0–20 nM) of TBP. (d) Regulation of Mango III aptamer fluorescence mediated by Myc-Max-Translator (right) in the presence of different concentrations (0–20 nM) of Myc-Max.

### Artificial Regulation of CRISPR-Cas13 via TF-Driven Cas12a Activity

To further demonstrate the platform’s capability of supporting synthetic biology networks, we extended TF-induced activation to a second protein complex. In this context, the establishment of protein circuits can play a key role in advancing synthetic biology, offering significant potential for the programmable control of biochemical and cellular processes.^79–81^ In this case, we selected CRISPR-Cas13a to establish a communication network with TBP and Cas12a (Figure 7a). Cas13a is part of the CRISPR-associated programmable endonucleases and can be directed by crRNAs, providing a platform for specific RNA sensing. Upon recognizing its RNA target, activated Cas13a engages in collateral cleavage of nearby nontarget RNA reporters, making it a powerful tool for nucleic acid diagnostics.^82,83^ In our approach, we utilized a single-stranded DNA blocker that, upon hybridization to the crRNA, prevents the formation of the ribonucleoprotein complex between RNA and Cas13a, thereby inhibiting its trans-cleavage activity. The formation of the heteroduplex creates a series of DNA bulges that can be cleaved by the TBP-mediated trans-cleavage activity of Cas12a. This would release the crRNA, allowing it to form the ribonucleoprotein complex with Cas13a, recognize its target, and cleave nearby FRET-labeled RNA hairpin reporters, generating an amplified fluorescence signal (Figure 7b). The fluorescence kinetic profiles in Figure 7b illustrate the results of TBP/Cas12a-mediated regulation of Cas13a trans-cleavage activity. The red and gray holey dots represent the activity of Cas13a in the presence and in the absence of the DNA blocker, respectively. To remove the blocker, we exploited TBP-mediated activation of Cas12a trans-cleavage by using TBP concentrations ranging from 7.5 to 15 nM. With a concentration of 15 nM, we successfully restored Cas13a activity within 120 min (Figure 7b). To confirm TBP-mediated regulation of Cas13a trans-cleavage activity, we conducted experiments in the absence of TBP or the TBP-Translator. These experiments showed no significant difference from the signal of blocked Cas13a (Figure S8), further validating our approach of successfully connecting three different non-naturally related biomolecular entities.

**Figure 7:**
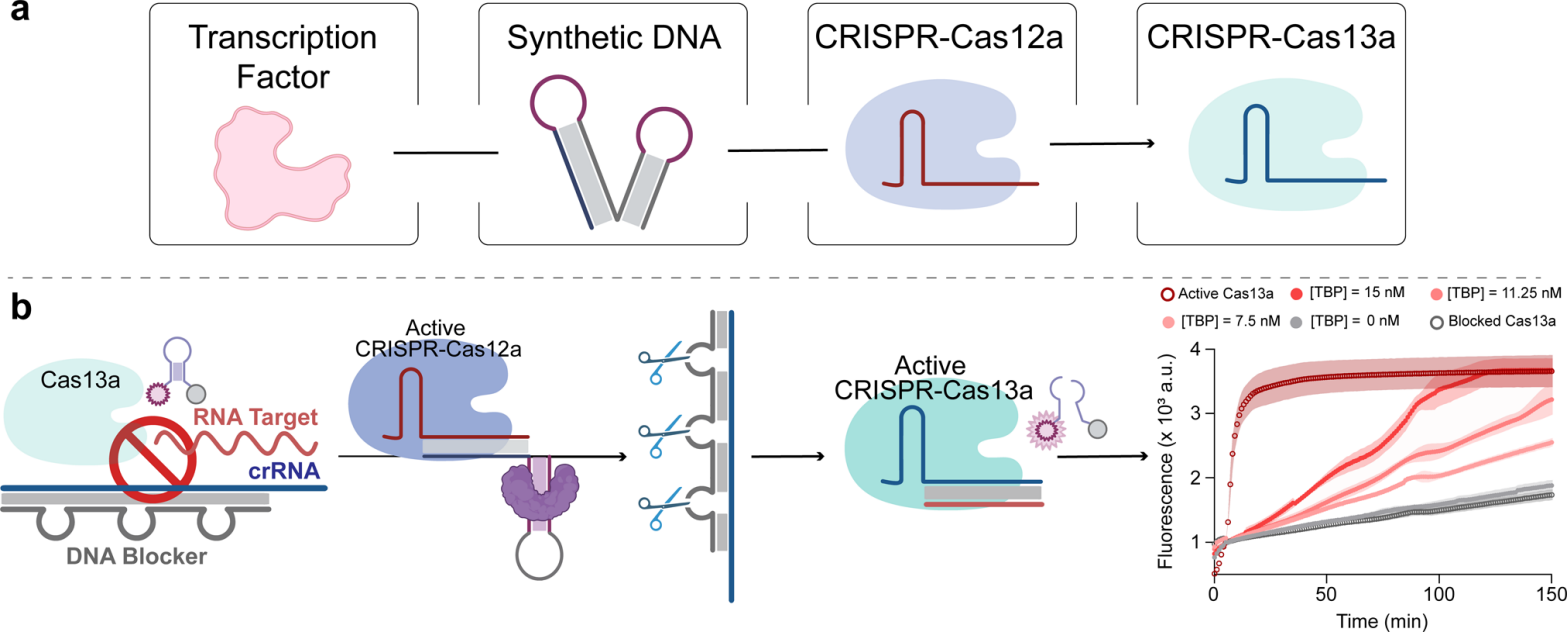
(a) Schematic representation of the molecular network modulated by the interaction between a transcription factor and its DNA translator to regulate CRISPR-Cas12a activity for the subsequent activation of CRISPR-Cas13a. (b) Schematic representation and fluorescence kinetic profiles of Cas13a activation controlled by TBP-induced CRISPR-Cas12a trans-cleavage activity.

## Conclusions

In this study, we successfully developed and characterized TF-responsive DNA translators that enable precise, programmable regulation of CRISPR-Cas12a activity. By leveraging the natural DNA-binding properties of TFs, we demonstrated the ability to regulate Cas12a trans-cleavage activity through engineered DNA translators that shift between conformations in response to TF binding. Our approach highlights the utility of transcription factors as protein-based regulators of CRISPR-Cas systems, thereby broadening their applications beyond traditional nucleic acid targets. Our work includes the optimization of different DNA translator designs, such as the Right Tail Design (RTD) and Left Tail Design (LTD), to achieve high signal gain and efficient kinetic performance. The TBP-Translator and Myc-Max-Translator were shown to provide TF concentration-dependent control of Cas12a activity with high specificity. Furthermore, we demonstrated the versatility of this system by integrating TF-mediated regulation of Cas12a activity into more complex synthetic biology networks such as downstream activation of the Mango III RNA aptamer and CRISPR-Cas13a. Notably, we demonstrated that artificial communication can be established between non-naturally related biomolecular species such as transcription factors, CRISPR-Cas12a and CRISPR-Cas13a by creating consecutive DNA-based artificial inputs. These results underscore the potential of our platform to enable future applications in biosensing, diagnostics, and synthetic biology. By translating protein presence into nucleic acid signals compatible with CRISPR machinery, our approach enriches the synthetic biology toolbox and paves the way for more complex biomolecular networks.

## Supplementary Material



## Notes

Materials and methods, DNA and RNA sequences, and supporting figures for specificity assays, RNA Mango system, and CRISPR-Cas13 activation (PDF)
