# Supplementary material for: Synthetic CRISPR Networks Driven by Transcription Factors via Structure-Switching DNA Translators
Source: J Am Chem Soc. 2025 Jun 10;147(24):21184–93. doi: 10.1021/jacs.5c06913 (PMC12186477; doi:10.1021/jacs.5c06913)
Supplement: Supplementary file 1 [file ja5c06913_si_001.pdf]

Supporting Information

Synthetic CRISPR Networks Driven by Transcription Factors via Structure-Switching DNA Translators

Luca Capelli, Sofia Marzari, Elena Spezzani, Alessandro Bertucci*

Department of Chemistry, Life Sciences and Environmental Sustainability, University of Parma, Parco Area Delle Scienze 17/A, 43124, Parma, Italy

Supporting Information

1. Materials and Methods

2. Supporting Figures

3. Supporting Tables

1. Material and Methods

1.1 Chemicals

PBS buffer solution was purchased from Corning (Manassas, VA, USA). $MgCl_2$, KCl and $ZnCl_2$ were purchased from Sigma Aldrich (Merck KGaA, Darmstadt, Germany). Tris (hydroxymethyl)-aminomethane was purchased from Tokyo Chemical Industry (Tokyo, Japan). TO1-3PEG-Biotin was purchased from abm (Applied Biological Materials, Richmond, BC, Canada).

1.2 Proteins

EnGen Lba Cas12a (Cpf1) was purchased from New England BioLabs (Ipswich, MA, USA). TATA-binding protein was purchased from Abcam (Cambridge, UK). Human Early Growth Response 1 (EGR1) protein was purchased from Abbexa (Cambridge, UK). Human Early Growth Response 4 (EGR4) protein was purchased from Abbexa (Cambridge, UK). Recombinant c-Myc/MAX Complex was purchased from Active Motif (Carlsbad, CA, USA). LwCas13a was purchased from Signalchem Diagnostics (Richmond, BC, Canada)

1.3 Oligonucleotides

The DNA sequences were designed and analyzed using the NUPACK software (www.nupack.org) and were purchased from Metabion international AG (Planegg, Germany). All oligonucleotides were dissolved in PBS pH 7.4 at a concentration of 100 µM and stored at -20 °C.

DNA and RNA strand sequences are reported in Table 1-7:

Table 1. crRNA

**crRNA Cas12a**	UAAUUUCUACUAAGUGUAGAUAUUCUGUGAAAUAAAGGUAA
**crRNA Cas13**	GGGAUUUAGACUACCCCAAAAACGAAGGGGACUAAAACUCAACAUCAGUCUGAUAAGCUA

Table 2. Reporter strand

**Reporter strand Cas12a**	(6-FAM)-CTCTCATTTTTTTTTAGAGAG-(BHQ1)
**Reporter strand Cas13**	(CY3)-CUC UCA UUU UUA GAG AG-(BHQ2)

Table 3. DNA translator for TBP

TBP-Translator Right Tail	
**TBP-Translator 1**	TACCTTAATATAAATAGGTTAGGTAATATTTATATTATTACCTTTATTTCACAGAAT
**TBP-Translator 2**	TACCTAAATATAAATAGGTTAGGTATAATTTATATTTATACCTTTATTTCACAGAAT
**TBP-Translator 3**	TACCTTAATATAAATAGGTTAGGTAAAATTTATATTTTTACCTTTATTTCACAGAAT
**TBP-Translator 4**	TAAATTTATATAAACATTTTAGGTAAGGTTTATATCCTTACCTTTATTTCACAGAAT
**TBP-Translator 5**	TACCTTTATATAAATAGGTTAGGTAAAATTTATATTTTTACCTTTATTTCACAGAAT

TBP-Translator Left Tail	
**TBP-Translator I**	TTACCTTTATTTCACAGAATAAATATATAAGGTTCACTCTATATTTTTAGTGT
**TBP-Translator II**	TTACCTTTATTTCACAGAATGAAATATATAAGGTTCACTATATATTTCTTAGTCT
**TBP-Translator III**	TTACCTTTATTTCACAGAATGAAATATATAAGGTTCACTATATATTTCCATAGTCT
**TBP-Translator IV**	TTACCTTTATTTCACAGAATGAAATATATAAGGTTAACTATATATTTCCTTAGTTA
**TBP-Translator V**	TTACCTTTATTTCACAGAATGAAATATATAAGGTTCACTATATATTTAATAGTCT

Underlined: TBP consensus sequence.

Table 4. DNA translator for Myc-Max

**Myc-Max Translator**	TTACCTTTATTTCACAGAATATGAATTAATGGTGCACTCTGACGTGCACCAGTCAGAGT<br>GT

Underlined: Myc-Max consensus sequence.

Table 5. Inhibitor sequences

**TBP inhibitor**	ATATAAACCCCCTTTATAT
**Myc-Max inhibitor**	CACGTGGTTTTACCACGTG

Table 6. Mango system

**Mango RNA Aptamer**	CGCGAUGGAAGGAUUGGUAUGUGGUAUAAUCGCG
**Mango DNA blocker**	ATCCTTCTTTTTTTTTCATCGCG

Table 7. Cas13 system

**Target RNA strand**	UAGCUUAUCAGACUGAUGUUGA
**Blocker DNA Strand**	GTTTTAGTCCTTTTTTTTTTCCTTCGTTTTTTTTTTTTTTTGGGGTAGTTTTTTTTTTT<br>C<br>TAAATCCC

1.4 Design of the DNA Translators

Different transcription factor-responsive DNA translators were engineered as double stem-loop hairpin structures in which the two loops encode the double stranded consensus sequence recognized by the transcription factor and one of the two stems incorporates the DNA activator sequence for CRISPR-Cas12a. This structure is in thermodynamic equilibrium with another mutually exclusive single-stem-loop hairpin conformation in which the stem is the full double stranded consensus sequence for the corresponding transcription factor and the DNA activator sequence is free to trigger the *trans*-cleavage activity of Cas12a. For each DNA translator, the predicted standard free energies of the two switching conformations were determined *in silico* using the OligoAnalyzer® and UNAFold web tools, setting T = 37° C, [Na+] = 200 mM, [Mg2+] = 5 mM. Based on these values, it was possible to calculate the predicted intrinsic switching equilibrium constant (K_s) of each DNA translator using the standard thermodynamic equation $\Delta G = -RT \cdot \ln(K)$ (Table S1).

1.5 Formation of Cas12a-crRNA Complex

To form the Cas12a-crRNA complex, a solution containing Cas12a and crRNA was prepared and then brought to a final volume of 20 µL with PBS, achieving a final concentration of 200 nM for both components. The solution was placed in a dry block thermostat set to 37°C for a 30-minute incubation period.

1.6 DNA Translator-Protein Complex Incubation

A solution in PBS with 5 mM $MgCl_2$ (PBS/Mg) was prepared containing 1:10 of DNA Translator:Target Protein, with a final volume of 24 µL. The solution was placed in a dry block thermostat set to 37°C for a 30-minute incubation period.

1.7 Fluorescence Measurements

A volume of 20 µL of the solution containing the DNA translator-protein complex was added to the solution containing the Cas12a-crRNA complex. This solution was then brought to a total volume of 100 µL with PBS/Mg. Finally, the reporter strand was added at a concentration of 200 nM.

Fluorescence measurements were carried out at λ_{exc}= 488 nm and λ_{em}= 520 nm for two hours (right-tail translator) or one hour (left-tail translator). All measurements were performed at 37°C.

Kinetic acquisitions were carried out using a Fluoromax-3 (150W continuous Xe source; excitation monochromator 200-950 nm; emission monochromator 200-950 nm; "Photon counting" emission detector with photomultiplier optimized for 290-850 nm; automatic Glan Thompson polarizers;

cuvette: Ultra-Micro Cell 10x2 mm, volume 100 µl, center height 15 mm, purchased from Hellma Analytics).

The fluorescence intensities recorded in the presence and absence (Background) of the protein were used to calculate the signal gain % (calculated with the following formula: signal gain % = (Fluorescence Signal – Background) / Background * 100, where the Background is the signal observed when conducting the assay in the absence of the protein).

1.8 Protein Concentration Assays

The concentration-dependent assays were conducted to measure the signal variations in response to different transcription factor concentrations. The experimental protocol was the same as that described above, using DNA translator at 2 nM and varying the concentration of the transcription factor in the working solution (0.02-100 nM for TBP, and 2-200 nM for Myc-Max). Plots of signal gain % vs concentration of TF were fitted with the following four parameters logistic equation:

$$Signal\ Gain\ \% = B_{min} + (B_{max} - B_{min}) \frac{[TF]^{n_H}}{K_{\frac{1}{2}}^{n_H} + [TF]^{n_H}}$$

Where Bmin and Bmax are the minimum and maximum signal gain % values, respectively; K1/2 is the concentration of the target at a half-maximum signal gain %, [TF] is the TF concentration, and nH is the Hill coefficient.

1.9 Specificity Tests

Specificity tests were conducted under the same conditions described above (section 1.7), using different transcription factors (2 nM) instead of the specific one associated with the DNA translator.

1.10 Inhibition Tests

To achieve protein activity inhibition mediated by an inhibitor strand, the following procedure was followed: first, a solution in PBS/Mg containing the protein and the inhibitor strand (1:10 protein:inhibitor) was pre-incubated at 37 °C. After 30 minutes, the DNA translator was added, and the solution was incubated for another 30 minutes. Meanwhile, in a separate tube, the Cas12a-crRNA complex was incubated for 30 minutes. From this point onward, the protocol is the same as that described above in section 1.7.

Background fluorescence measurements were performed by incubating the inhibitor strand with the DNA translator in the absence of the protein. This solution was then added to the one containing the CRISPR-Cas12a complex, as described previously.

1.11 RNA Mango System

A solution was prepared by adding the RNA Mango Aptamer and 1.2-fold molar excess of the Mango inhibitor strand in 20 µL of PBS. Following this, annealing was performed. The Cas12a-crRNA and DNA translator-protein complexes were formed as previously described. After combining the two complexes in a test tube, 20 µL of the solution containing the Mango-inhibitor complex was added. The reaction was allowed to proceed for 5 minutes, after which 4 M KCl and 65 µM TO-1 were added, and a fluorescence spectrum was subsequently recorded (λ_{exc}= 505 nm and λ_{em}= 515-580 nm) on a FP-*8259 Jasco Inc* (Hachioji, Tokyo, Japan) fluorometer.

1.12 Formation of Cas13a-crRNA Complex

Cas13a and crRNA were combined in a 1:1 molar ratio in PBS containing 5 mM $MgCl_2$ to achieve a final concentration of 1 µM. The mixture was incubated at 37°C for 30 minutes in a dry block thermostat.

1.13 Assembly of the crRNA-Blocker Heteroduplex

The crRNA-blocker heteroduplex was prepared in PBS with 5 mM $MgCl_2$, using a 1.2-fold molar excess of the blocker. The reaction mixture was subjected to a thermal ramp, heating from room temperature to 60°C, followed by gradual cooling to promote heteroduplex formation.

1.14 DNA Translator-Protein Complex Incubation

A solution in PBS with 5 mM $MgCl_2$ (PBS/Mg) was prepared containing 40 nM DNA translator and varying concentrations of TBP. Specifically, DNA translator-to-TBP ratios of 1:10, 1:15, and 1:20 were tested. The solution was incubated at 37°C for 30 minutes in a dry block thermostat. Background fluorescence measurements were performed under identical conditions but without the addition of TBP.

1.15 Fluorescence Monitoring

The crRNA-blocker mixture was then transferred to a cuvette, and Cas13 target was added to a final concentration of 11 nM, along with Cas13 enzyme (66 nM) and Cas13 reporter (200 nM). The pre-formed crRNA-Cas12 complex was added to the reaction to reach a final concentration of 15 nM. Fluorescence was monitored by measuring Cy3 emission (λ_exc = 538 nm, λ_em = 564 nm) for 150 minutes at 37°C. After 5 minutes, the TBP-translator complex was introduced, achieving a final concentration of 0.75 nM for the DNA translator. Kinetic acquisitions were carried out using a FP-*8259 Jasco Inc* (Hachioji, Tokyo, Japan) fluorometer.

1.16 Data Analysis

Data plotting and statistical analysis were performed using Prism8 (GraphPad). The experimental results presented in the bar graphs represent the mean of three independent replicates plus the standard deviation (SD).

2. Supporting Figures

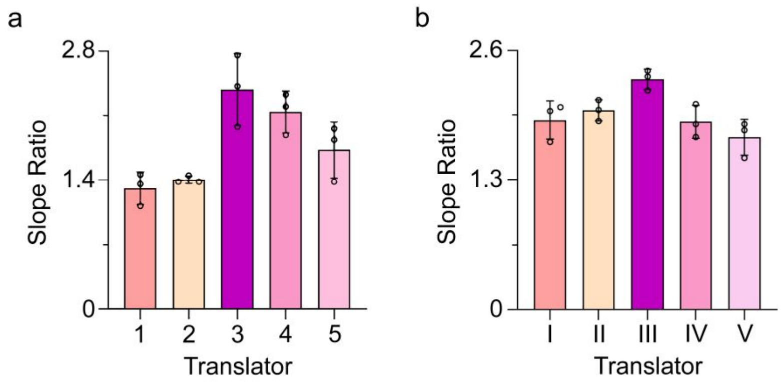

Figure S1. Calculated ratio between the slopes of the initial fluorescence kinetic curves for Cas12a activity in the presence and absence of TBP. a) RTD Translators. b) LTD Translators.

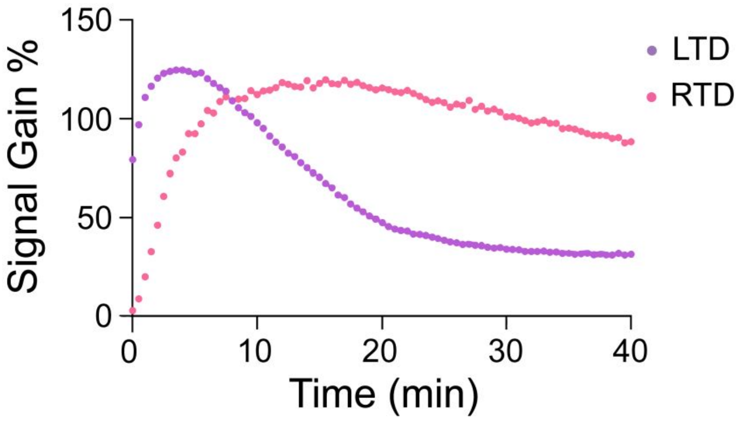

Figure S2. Comparative analysis of Signal Gain % as a function of time within the first 40 minutes of Cas12a activity for LTD and RTD.

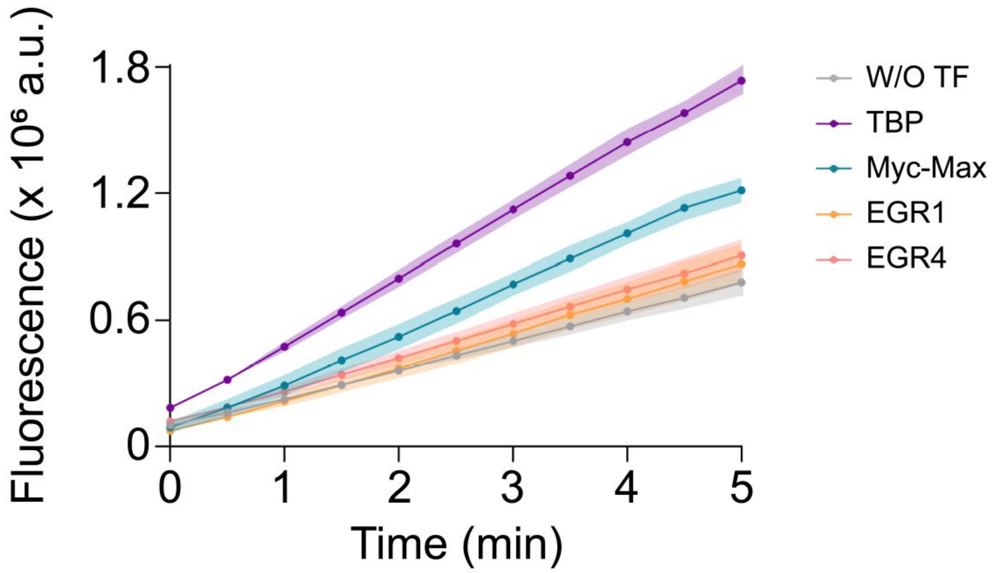

Figure S3. Fluorescence kinetic profiles obtained when the TBP-Translator/CRISPR-Cas12a system is incubated with target TBP or non-target TFs such as Myc-Max, EGR1 and EGR4.

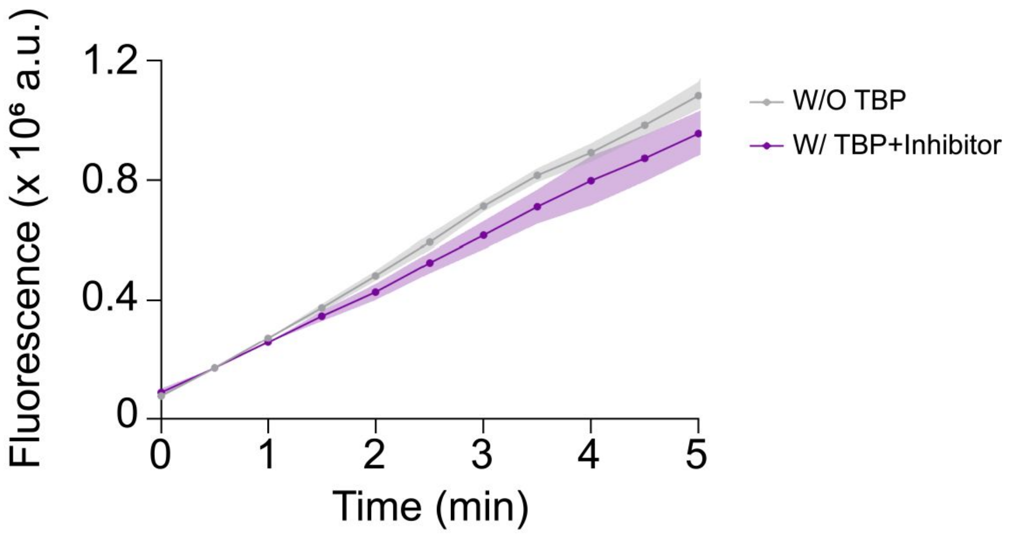

Figure S4. Fluorescence kinetic profile obtained when the TBP-Translator/CRISPR-Cas12a system is incubated with TBP in the presence of a TBP inhibitor.

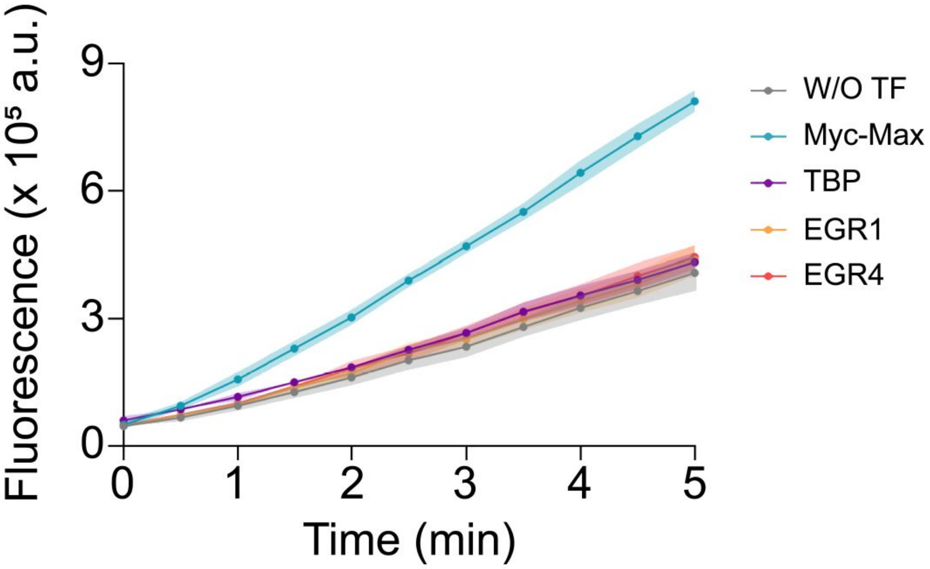

Figure S5. Fluorescence kinetic profile obtained when the Myc-Max-Translator/CRISPR-Cas12a system is incubated with target Myc-Max or non-target TFs such as TBP, EGR1 and EGR4.

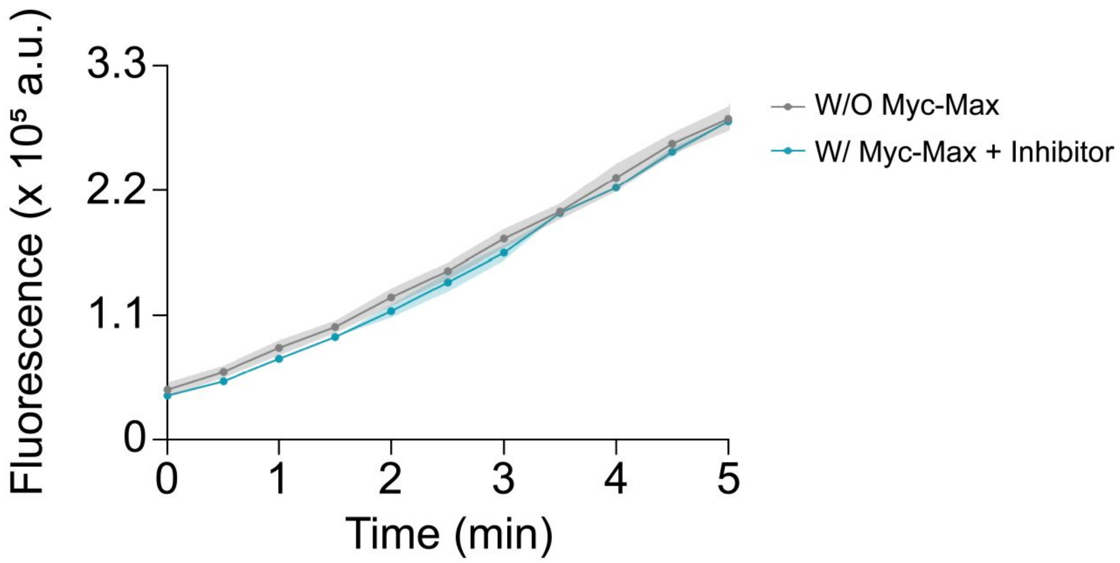

Figure S6. Fluorescence kinetic profile obtained when the Myc-Max-Translator/CRISPR-Cas12a system is incubated with Myc-Max in the presence of a Myc-Max inhibitor.

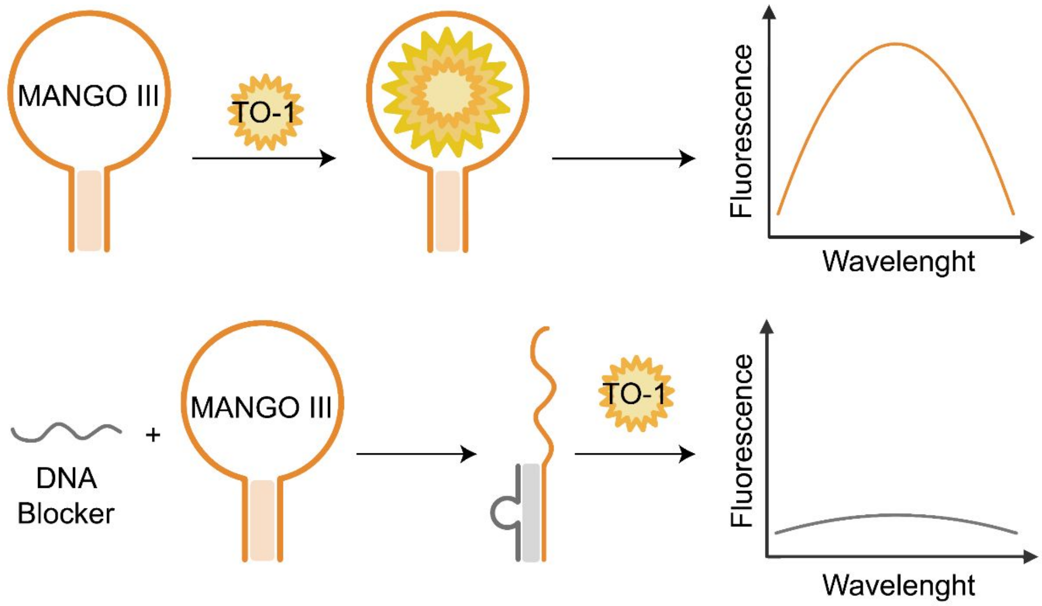

Figure S7. Schematic representation of Top) Mango III aptamer functioning, and Bottom) the proposed blocking strategy.

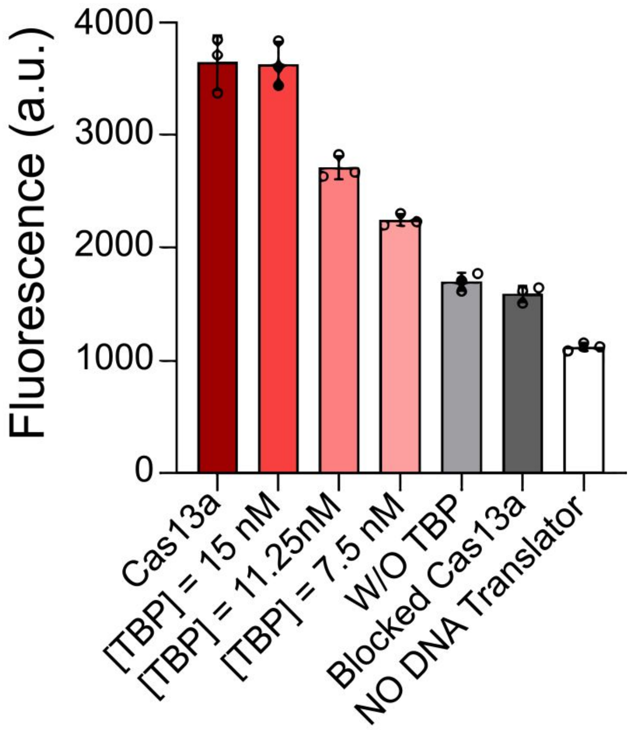

Figure S8. Fluorescence intensity observed for Cas13 trans-cleavage activity in the different conditions reported in the graph.

3. Supporting Tables

Ks Values	TBP-Translator 1	TBP-Translator 2	TBP-Translator 3	TBP-Translator 4	TBP-Translator 4
**OligoAnalyzer®**	0.23	0.079	0.023	0.014	0.005
**UNAFold web**	0.23	0.079	0.023	0.014	0.005

Table S1. Comparison between Ks values calculated with OligoAnalyzer® and with UNAFold web.
